# Galectin-3 Determines Tumor Cell Adaptive Strategies in Stressed Tumor Microenvironments

**DOI:** 10.3389/fonc.2016.00127

**Published:** 2016-05-23

**Authors:** Ana Carolina Ferreira Cardoso, Luciana Nogueira de Sousa Andrade, Silvina Odete Bustos, Roger Chammas

**Affiliations:** ^1^Departamento de Radiologia e Oncologia, Faculdade de Medicina, Centro de Investigação Translacional em Oncologia, Instituto do Câncer do Estado de São Paulo, Universidade de São Paulo, São Paulo, Brasil

**Keywords:** galectin-3, cell signaling, migration, angiogenesis, tumor microenvironment

## Abstract

Galectin-3 is a member of the β-galactoside-binding lectin family, whose expression is often dysregulated in cancers. While galectin-3 is usually an intracellular protein found in the nucleus and in the cytoplasm, under certain conditions, galectin-3 can be secreted by an yet unknown mechanism. Under stressing conditions (e.g., hypoxia and nutrient deprivation) galectin-3 is upregulated, through the activity of transcription factors, such as HIF-1α and NF-κB. Here, we review evidence that indicates a positive role for galectin-3 in MAPK family signal transduction, leading to cell proliferation and cell survival. Galectin-3 serves as a scaffold protein, which favors the spatial organization of signaling proteins as K-RAS. Upon secretion, extracellular galectin-3 interacts with a variety of cell surface glycoproteins, such as growth factor receptors, integrins, cadherins, and members of the Notch family, among other glycoproteins, besides different extracellular matrix molecules. Through its ability to oligomerize, galectin-3 forms lectin lattices that act as scaffolds that sustain the spatial organization of signaling receptors on the cell surface, dictating its maintenance on the plasma membrane or their endocytosis. Galectin-3 induces tumor cell, endothelial cell, and leukocyte migration, favoring either the exit of tumor cells from a stressed microenvironment or the entry of endothelial cells and leukocytes, such as monocytes/macrophages into the tumor organoid. Therefore, galectin-3 plays homeostatic roles in tumors, as (i) it favors tumor cell adaptation for survival in stressed conditions; (ii) upon secretion, galectin-3 induces tumor cell detachment and migration; and (iii) it attracts monocyte/macrophage and endothelial cells to the tumor mass, inducing both directly and indirectly the process of angiogenesis. The two latter activities are potentially targetable, and specific interventions may be designed to counteract the protumoral role of extracellular galectin-3.

## Galectin-3 and Its Key Structure to Function Relationships

Besides the common sense that most of the biological information resides in both nucleic acid and amino acid sequences, glycans present either on the cell surface or in the extracellular matrix (ECM) molecules also constitute an important reservoir of biological information. The coding capacity of carbohydrates is much broader than DNA sequences and peptides due to the structural and conformational diversity displayed by these molecules (1). This so-called “sugar code” collectively defines boundaries within tissues, thus serving as a constitutive territorial marker, besides indicating transient states of the cells present within these tissues. Different animal lectins, glycan-binding proteins, decipher the biological information imprinted in the “sugar code” and trigger signaling pathways, orchestrating a variety of biological and pathophysiological processes.

Animal lectins are categorized into structurally related families and superfamilies, based on highly conserved carbohydrate recognition domains (CRDs) (2). Within the lectin superfamilies, galectins constitute a family of animal lectins known to mediate a plethora of processes like cell adhesion, migration, survival, death, and differentiation. Initially termed S-type lectins, galectins are a group of proteins expressed in all organisms. Galectins are characterized to bind β-galactose-containing glycoconjugates and share primary structural homology in their CRDs (3). This family, composed by 15 mammalian galectins, is classified in three subtypes or groups, namely (i) prototype, (ii) chimera, and (iii) tandem repeat group, according to the number and organization of CRDs (4).

Galectin-3 is the only member of the chimera group (5). Galectin-3, initially known as Mac-2, ϵBP, CBP35, CB-30, and L29 among another names, is one of the best studied lectins and is constituted by three distinct domains: an NH2-terminal domain, a proline-rich collagen-α-like domain, and a COOH-terminal domain that contains the CRD (6). This protein is encoded by a single gene in humans (*LGALS3*) composed of five introns and six exons located in chromosome 14, locus q21–q22, and the protein has a molecular weight of ~31 kDa (7, 8). Its promoter region, as well as its first exon, exhibits a high content of CpG islands, indicating that epigenetic mechanisms also control galectin-3 expression, as observed during malignant transformation and tumor progression (9).

Members of the galectin family bind simple β-galactosides, such as disaccharides or trisaccharides. However, this affinity is relatively weak compared to binding to natural glycoconjugate ligands expressed on cell surfaces or in the ECM. Galectin CRDs recognize different types of glycan ligands and show high affinity binding to different structures. Galectin-3 was originally described to bind preferentially to type 1 or type 2 Galβ1→3(4)GlcNAc (*N*-acetyllactosamine) chains, and its affinity increases for polylactosamine structures and/or branched glycans over simple saccharides (10) (For more information, access “Consortium for Functional Glycomics,” Paradigm pages, accessed February 22, 2016, http://www.functionalglycomics.org). However, specific binding of galectin-3 may be attenuated or enhanced, depending on the substituents that modify subterminal galactose residues in the galectin ligand. Accordingly, galectin-3 shows higher affinity to oligosaccharides bearing 2- or 3-O-α-substituents on the outer galactose residue of glycans, such as NeuNAcα2,3 lactosamine or the A-blood group structure GalNAcα1,3 [Fucα1,2]Galβ1,4GlcNAc (11). Additionally, sialylation and sulfation of glycoconjugates also interfere on the carbohydrate affinity and the specificity of galectins. For example, presence of 3′-*O*-sulfated or 3′-*O*-sialylated glycoconjugates increases the affinity toward galectin-8 (12). Otherwise, 6-O-α-substitution by sialic acid reduces galectin-3 carbohydrate affinity (13–16). Suzuki and colleagues have also demonstrated that pretreatment of H-ALCL cells with neuraminidase, which cleaves cell surface sialic acid, increases galectin-3 adhesion to integrins (17).

Carbohydrate-binding characteristics of galectins involve (i) the array of glycan ligands; (ii) the architecture, dynamics, and binding sites of CRDs; and (iii) the topological display of glycans. It has been suggested that differences in the amino acid properties of galectins are responsible for the different binding of glycans to the CRDs, since the 3D structure of the galectin CRDs have an almost identical fold, while their amino acid sequence identity is rather low (18–20). For example, the CRD of galectin-3 is 30–40% identical with galectins 4–10 and 20–25% identical with galectin-1 and -2 (21–23). These differences provide specific structural features that determine carbohydrate-binding specificity. For example, galectin-3 has a higher affinity for GalNAc than galectin-1, due to an arginine residue at position 144 (23). The topological display of glycan ligands in glycoconjugates (glycoclusters) is also important to an appropriate spatial match between lectin domains and polyvalent ligands (24, 25). Upon binding, galectins may form supramolecular structures due to the formation of homodimers and multimers. Altogether, the binding characteristics of each galectin and their ability to form higher order complexes with their ligands will determine the biological functions ascribed to galectins.

The relationship between the quaternary structure and galectin-3 precipitation activity also provides information about its structure-bioactivity and binding properties. There are two mechanisms for the formation of oligomers of galectin-3; an N-terminal-dependent and a C-terminal-dependent association (26). The N-terminal-dependent association of galectin-3 was the first oligomerization mechanism described (27–30). In solution, monomeric galectin-3 is in equilibrium with oligomers and may precipitate as a pentamer in the presence of multivalent oligosaccharides (31–35). The N-terminal domains confer oligomer flexibility, making the overall oligomer highly flexible, which contribute to galectin-3 ability to dynamically convert to pentamers that form heterogeneous cross-linked complexes with specific multivalent glycoconjugates (33). The capacity of forming multivalent oligomers is responsible for the formation of lattices on plasma membranes, which contribute to stability and the biological functions of galectin-3 (36). This property of galectin-3 is targetable. For example, galectin-3C, a truncated form of human galectin-3, functions as an inhibitor of galectin-3 oligomerization and displays a more effective antitumor activity than the intact lectin (37–39).

Galectin-3 lattices are high-order supramolecular structures of the lectin cross-linked with its specific ligands. The most studied galectin-3 ligands are found in *N*-glycans from cell surface or ECM glycoproteins. The amount of *N*-glycan structures in a given glycoprotein determines the probability of lattice formation, which in turn interferes with biological aspects of the glycoprotein function, such as its endocytosis (receptor turnover) or its ability to transduce signals (40). Through its ability to form glycoprotein lattices, galectin-3 tunes the function of different receptors, such as the epidermal growth factor receptor, platelet-derived growth factor receptor, fibroblast growth receptor, vascular endothelial growth factor receptor (VEGFR), and transforming factor-β receptor (40–42). It is worth noting that galectin-3 is not always associated with activation of endocytosis of glycoprotein receptors. Indeed, a galectin-3-dependent lattice can lead to persistence of a given receptor on the cell surface. For example, this observation suggests that galectin-3 does not directly determine the process of endocytosis but will be determined by the partners of the galectin-3 ligand on the cell surface.

Galectin-3 is widely distributed among different species and, at the cellular level, it can be found in the extracellular and intracellular milieu (43–45). Hence, galectin-3 can participate in many important biological processes in the cytoplasm and nucleus, where no cognate carbohydrate ligands for galectin-3 are often present. These functions result from protein–protein interactions, showing the versatility of this molecule. Accumulating evidence shows that galectin-3 interacts with a large number of molecules. Based on the distribution of galectin-3 in different subcellular compartments and its ability to interact with different molecules, it has been suggested that galectin-3 serves as a shuttling protein (46), playing distinct roles in many biological processes depending on its localization. In the nucleus, galectin-3 modulates cell survival and mRNA splicing (47, 48). For example, galectin-3 forms complexes with members of the spliceosome mediating pre-mRNA splicing and spliceosome assembly in a carbohydrate-independent manner (49). Despite the absence of glycan ligands in intracellular compartment, electrostatic potential calculations show a linear array of three positively charged arginine residues in a cleft of galectin-3 CRD that suggests a possible binding site to RNA (23). In the same context, intracellular galectin-3 CRD modulates DNA damage response through interacting with BARD1, a BRCA1 partner (50), and attenuates the pro-apoptotic activity of Bax through interaction with members of the bcl-2 family (51–55).

Galectin-3 undergoes posttranslational modifications, such as limited proteolysis and phosphorylation. Extracellular galectin-3 may be cleaved by different molecules, such as matrix metalloproteases (MMPs) and the prostate-specific antigen (PSA). MMP-dependent processing leads to its truncated form. As mentioned above, this truncated form of galectin-3 interferes with galectin-3 multimerization and biological functions. Structural variants of galectin-3, due to single nucleotide polymorphisms or phosphorylation at specific sites of full galectin-3, interfere with proteolysis. There are three different single nucleotide polymorphisms in galectin-3 gene distributed in human populations (56, 57). For example, the change Pro64 for His64 has been associated with alterations in galectin-3 function and with breast (58) and prostate cancer (59) incidence. In fact, this P64H substitution is susceptible to MMP-2 and -9 cleavages (60).

Phosphorylation implicates conformational changes of the protein, altering the interaction of galectin-3 with ligands and its ability to participate in multivalent interactions (61–63). Although the physiological significance of this posttranslational modification remains unclear, it has been suggested that phosphorylation of galectin-3 may modulate its intracellular function and translocation. Galectin-3 may be phosphorylated at N-terminal Ser (Ser6 and Ser12) or at Tyr sites (61, 64–66). For example, serine phosphorylation has been associated with differential binding to laminin and mucin, besides playing a role in nuclear export and antiapoptotic activity of galectin-3 (67–69). Tyrosine phosphorylation of galectin-3, mediated by c-Abl kinase, appears to be essential for cell motility, lysosomal degradation, cleavage inhibition, and secretion of galectin-3 (58, 70, 71). However, the mechanisms involved in galectin-3 secretion and its traffic among different organelles in the intracellular compartment are not well-understood. Galectin-3 is unlikely to be secreted by the ER/Golgi pathway since it lacks the classical secretion signal sequence. In fact, few studies suggest alternative mechanisms like vesicular release (72, 73), exosomes (74), and even a mechanotransduction-based mechanism (75) for its extracellular secretion. A conceivable possibility in pathophysiological conditions, including cancers, is that part of extracellular galectin-3 is released passively from dying cells. The basic mechanisms for galectin-3 subcellular compartmentalization have been partly unraveled. Galectin-3 lacks a nuclear localization sequence (NLS), and it interacts with components of the nuclear pore complex for its nuclear import–export (76–80). However, the fine control of galectin-3 translocation within subcellular compartments is not clearly understood.

Based on the aforementioned, it is not surprising that galectin-3 is also involved in many pathological conditions like cancer. Indeed, the importance of carbohydrates and lectins in cancer biology can be illustrated by the specific glycosylation signatures found in human tumors (81–84). Besides multiple factors like availability and localization of nucleotide sugar donors and substrates contributing to aberrant glycosylation in cancer, the main mechanism seems to be the differential expression of glycosyltransferases and glycosidases involved in the synthesis and catabolism of glycans (85). Besides changes in glycan sialylation, which interferes with galectin-3-binding as discussed above, cancer cells often present a significant increase in beta1–6 branching in *N*-linked glycans due to increased MGAT5 (UDP-*N*-acetyl-d-glucosamine: *N*-acetylglucosamine transferase V) activity (81, 82). MGAT5 overexpression is responsible to enhance metastasis potential in mouse mammary cancer cells (86). Canine mammary tumor cells entering the blood stream accumulate a large amount of galectin-3, which is associated with emboli formation (15, 87). Besides altered glycosylation, galectin-3 expression is commonly dysregulated within the tumor microenvironment, either by altered expression in tumor cells or by its expression in infiltrating leukocytes [for a comprehensive list of studies in human tumors, see Ref. (88)].

## Galectin-3 in Cancer Cell Signaling: A Role for Galectin-3 in the Tuning of Cellular Stress Signaling Pathways

**Figure 1:**
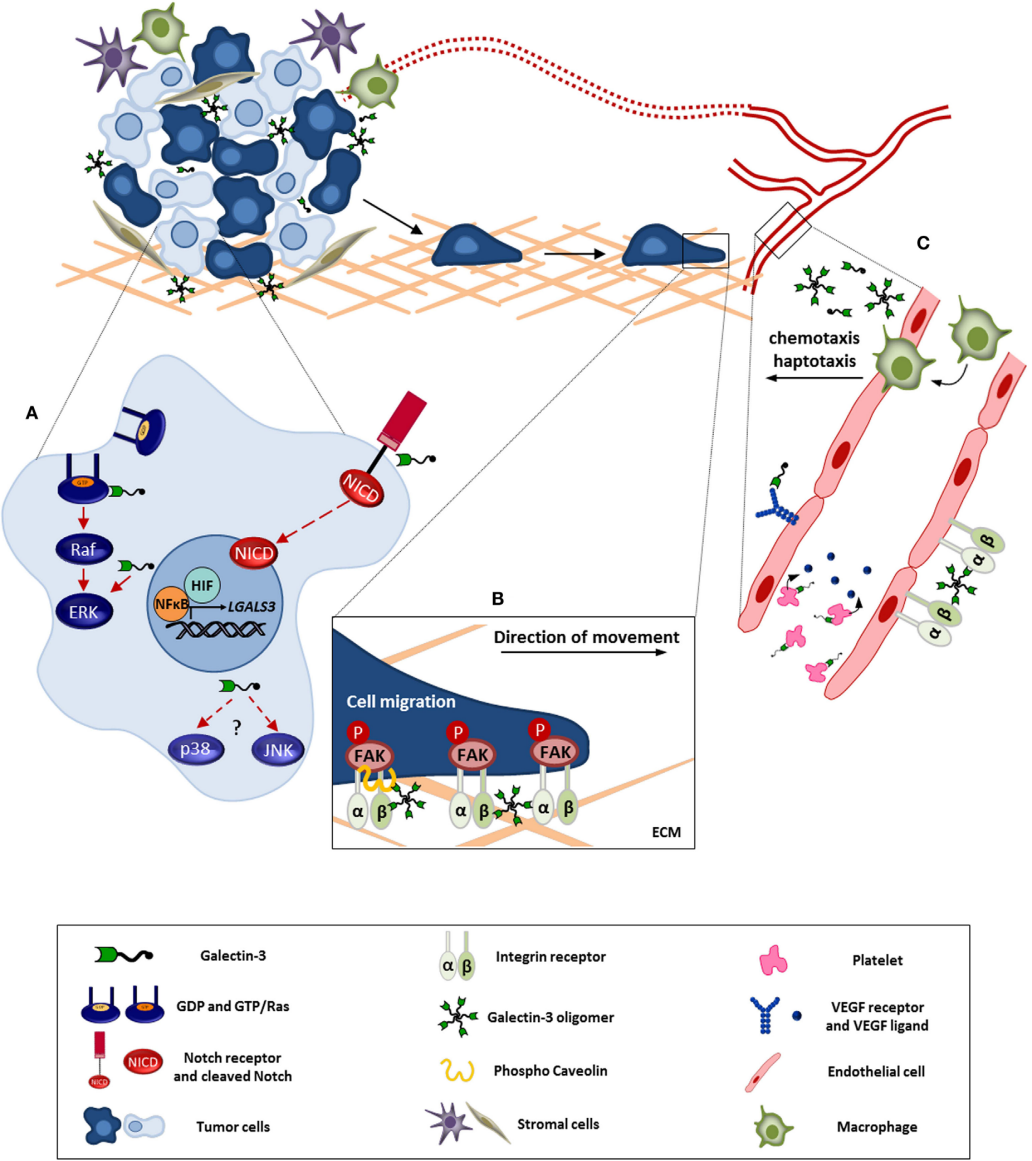
**Galectin-3 involvement in tumor progression**. This figure represents the intra-and extracellular galectin-3 functions in processes like cell survival, migration, and angiogenesis. **(A)** In tumor cell, galectin-3 regulates signaling pathways like, Ras/Raf/MEK/ERK and Notch, modulating the cell survival, proliferation, and migration. Besides, NFκB and HIF positively regulate galectin-3 expression contributing to its function within the tumor microenvironment. **(B)** Extracellular galectin-3 promotes tumor cell migration through interaction with mediators, such as integrins and caveolin, leading to FAK stabilization. **(C)** Regarding angiogenesis, the full-length galectin-3 can form oligomers and bind to endothelial cell surface, preventing VEGFR and integrin internalization. In addition, galectin-3 induces VEGF release by platelets. Furthermore, galectin-3 promotes monocyte/macrophage chemotaxis toward tumor microenvironment potentializing macrophage-induced angiogenesis. ECM, extracellular matrix. Red full arrows: galectin-3 contributes directly to pathway activation. Red dashed arrows: possible interaction of galectin-3 in described pathway.

It is well-documented that galectin-3 expression is altered in malignant tissues, and it has many functions in cancer progression, additionally the localization of this molecule is an important feature to understand its function, as already mentioned (84, 89). Here, we will discuss the involvement of galectin-3 in stress signaling pathways (MAPK family, from mitogen-activated protein kinase) and in the orchestration of both cell and tissue responses in stress conditions commonly found within the tumor microenvironment (Figure 1).

MAPKs are serine–threonine kinases that link extracellular signals with a diversity of cellular processes, such as growth, proliferation, migration, survival, and death (90). Abnormalities in MAPK signaling play a key role in development and progression of cancer. MAPK family consists of three main groups (i) extracellular signal-regulated kinase (ERK), which is activated by the Ras–Raf–MEK cascade; (ii) p38; and (iii) c-Jun NH_2_-terminal kinase.

Galectin-3 was first associated with Ras signaling in cancers, as galectin-3 interacts selectively through its CRD with activated K-Ras (K-Ras-GTP) and stabilizes it in the “on” state (91). Activated K-Ras enhances the translocation of galectin-3 to the plasma membrane and thereby galectin-3 increases K-Ras signaling, promoting phosphoinositide 3-kinase (PI3-K) activation and controlling both the intensity and duration of the K-Ras signal (Figure 1A). Through this mechanism, galectin-3 and Ras regulate important processes in tumor cells, such as proliferation and survival, playing key roles in different cancer cells (e.g., breast cancer cells). Galectin-3 is necessary for optimal activation of K-Ras/MEK pathway in breast cancer cells (92). It has been proposed that high expression of galectin-3 cooperates with K-Ras transforming activity, leading to the malignant phenotype. Additional data revealed that galectin-3 is an integral component of nanoclusters containing K-Ras, supramolecular structures partitioned into the plasma membrane. Formation of nanoclusters is essential for high fidelity signal transduction, with the ability to increase K-Ras activation to drive tumorigenesis *via* constitutive activation of Raf/MEK/ERK signaling cascade (93).

Galectin-3 is strongly expressed in thyroid carcinoma cells, but not in benign tumors, and as such, it is associated with the levels of GTP-bound K-Ras, thus contributing to thyroid carcinoma malignancy. Furthermore, the disruption of the interaction between Ras/galectin-3 reduced ERK activation, enhanced the cell cycle inhibitor p21 expression, and inhibited proliferation *in vitro* and tumor growth in nude mice (94). Song and colleagues investigated the effects of galectin-3 in downstream signaling events to the Ras pathway, using complementary *in vitro* and *in vivo* systems in pancreatic carcinogenesis. These studies showed that galectin-3 downregulation leads to decreased activation of AKT and ERK; thus, decreasing cell invasion and reducing tumor growth in an orthotopic mouse model (95). Moreover, in 2008, Saegusa and collaborators had reported that galectin-3 had an antiapoptotic role in keratinocytes treated with etoposide or irradiated with UVB light. In these experiments, keratinocytes devoid of galectin-3 were more susceptible to apoptotic stimuli by altered activation of ERK and reduced activation of AKT (96). The pro-survival role of intracellular galectin-3 and its association with the activation of Ras/Raf/MEK/ERK and the PI3-K/AKT pathways is now clear. Moreover, it has also been shown that galectin-3 enhances the migration of colon cancer cells (97) through activation of the K-Ras–Raf–ERK1/2 pathway (discussed below).

More recent studies have been carried out to understand the interaction of galectin-3 and downstream targets of the MAPK pathway. Gao and colleagues have investigated the roles and mechanisms of circulating galectin-3 in signal transduction, specifically in ERK signaling. Although intracellular galectin-3 increased ERK phosphorylation through RAS activation, Gao found that exogenous galectin-3 may stimulate positively ERK1/2 in a calcium-sensitive and PKC-dependent manner. Using truncated proteins, they demonstrated that intact extracellular galectin-3 is required to activate ERK1/2 in order to promote cell migration. In this study, AKT signaling was not activated by circulating galectin-3 (98). In another related study, it was observed that binding of galectin-3 to mucin 1 (MUC1), a mucin involved in potentiating growth factor-dependent signal transduction, enhances cell proliferation and motility in different epithelial cancer cells, through activation of both ERK1/2 and AKT pathways. Accordingly, galectin-3-depleted cells grew slowly as compared to the parental galectin-3-expressing cells (99). In sarcoma cells, galectin-3 disrupts focal adhesion plaques, inducing cell migration in an AKT-dependent manner (100).

There are few reports about galectin-3 and its relation with p38 or JNK kinases (Figure 1A). Both kinases are more responsive to stress stimuli than growth factors, comparing with ERK1/2. Thus, when Borges and collaborators investigated the effect of copper complexes in melanoma cells, they demonstrated that the metal treatment increased the levels of intracellular reactive oxygen species (ROS), which was accompanied by p38 activation in galectin-3-expressing melanoma cells (101). In addition, it has been reported that extracellular galectin-3 induces MMP-9 expression *via* p38 MAPK pathway in melanoma cells (102). These results show a different function of galectin-3 in p38 regulation, associated with another critical circuitry of the malignant phenotype, invasion, and metastasis. Regarding the JNK kinase family, it is known that intracellular galectin-3 activates elements of the JNK pathway. Evidence exists that the activation of both ERK and JNK1/2 depend on the phosphorylation status of the Ser^6^ residue of galectin-3. Phosphorylation of galectin-3 by kinases, such as casein kinase, seems necessary to the activation of antiapoptotic circuits dependent on ERK and JNK (68). Although it is known that galectin-3 regulates the activity of MAPK pathway in several cancer models, further studies are still necessary to elucidate the mechanistic details of the pro-survival activity of galectin-3. These studies will be necessary to design targeting strategies for potential clinical interventions.

### Galectin-3 Expression Is Regulated by Both NF-κB and HIF

NF-κB is a family of transcription factors that plays important roles in the immune system and regulates the expression of cytokines, cyclo-oxygenase 2 (COX-2), growth factors, and inhibitors of apoptosis. Moreover, pathological dysregulation of NF-κB is associated with inflammatory and autoimmune diseases as well as cancer (103). On studying galectin-3 expression in glioblastoma cells exposed to a variety of stressing stimuli, Dumic and colleagues found that NF-κB inhibition by specific proteasomal inhibitors decreased the expression of galectin-3 (104). In the same way, it was also showed that the interference in NF-κB activation can inhibit galectin-3 expression leading to apoptotic processes (105). We have found a relationship among galectin-3, NF-κB, hypoxia and nutrient deprivation, and common stressing conditions within the tumor microenvironment. HIF-1α is a master regulator of gene transcription under hypoxia, upregulating several genes, including galectin-3, in order to maintain cellular homeostasis and promote cell survival in skeletal tissues (106). At the tissue level, galectin-3 accumulates in hypoxic/nutrient-deprived areas from both glioblastoma and mammary tumors (87, 107–109). Under hypoxic conditions, galectin-3 transcription requires protein synthesis and depends on both HIF-1α and NF-κB activities (Figure 1A). Nuclear translocation of NF-κB was also induced within hypoxic/nutrient-deprived microenvironments, such as pseudopalisades in glioblastomas (109). Such areas are enriched in galectin-3, which protects cells from death. More recently, another study has shown that galectin-3 deficiency reduces proliferation of hepatoma cells and increases their rate of apoptosis both *in vitro* and *in vivo*. Conversely, it was demonstrated that galectin-3 expression induced by NF-κB transactivation led to a more invasive phenotype of tumor cells, which developed larger tumors as compared to those found in galectin-3^−/−^ mice (110).

### Galectin-3 and Notch Signaling

Notch signaling plays a key role in differentiation, survival, and/or proliferation. Alterations in the regulation of these processes contribute to malignant transformation – abnormal activation of this pathway is often found in various types of cancers. Notch signaling pathway involves communication among adjacent cells, one expressing a ligand (either Delta or Jagged) and the other expressing Notch, as a receptor and signal transducer (111). Initially, it was described that galectin-1 increased the expression of Notch1/Jagged2, promoting lung cancer progression (112). Fermino and collaborators then showed that endogenous galectin-3 selectively regulates downstream targets of Notch signaling pathway. Cells devoid of galectin-3 displayed a higher expression of Notch and its target gene *HES-1* in an infection model (113). Nakajima and colleagues had described a galectin-3-dependent activation of Notch1 signaling in a system that models tumor cell/osteoblast and osteoclast interactions, critical events in the maintenance of bone metastasis found in different cancers, such as breast and prostate cancers. Using coculture of human fetal osteoblasts (hFOB) with cancer cells expressing galectin-3, these authors showed that galectin-3 secreted by cancer cells inhibited osteoblast differentiation, a Notch1-regulated process. Extracellular galectin-3 interacted with Notch1, in a CRD-dependent manner, activating Notch through its proteolytic cleavage, which leads to the formation of Notch intracellular domain (NICD) with its subsequent translocation to the nucleus leading to Notch target genes upregulation (Figure 1A), which in turn is associated with the maintenance of a non-differentiated state of the osteoblast. In this set of experiments, Nakajima and colleagues also evaluated both intact galectin-3 and a truncated form of galectin-3 containing primarily its CRD. Although both forms of galectin-3 led to the activation of Notch, results shown apparently indicate that the intact galectin-3 is more efficient in the induction of Notch cleavage than its truncated form. Altogether, these events promoted suppression of osteoblast differentiation and caused bone remodeling in bone metastatic lesions (114). These experiments open a venue for research in a variety of Notch-dependent processes, from maintenance of a non-differentiated state of cells/tumor cells, activation of survival pathways, which in turn lead to resistance to different therapeutic strategies, to the process of angiogenesis.

## Galectin-3 Promotes Tumor Migration

Tumor progression is dictated by the bidirectional interaction between tumor and host cells that compose the tumor microenvironment. Besides that, it is also true that the ECM is an important non-cellular component of the tumor microenvironment that interferes with tumor initiation and progression. Based on the fact that galectin-3 can be secreted by cells (either actively or passively) and acts in the extracellular environment either as a monomer or a multimer, forming complexes with other molecules, one might expect that, upon interaction with its specific partners, galectin-3 interferes with different aspects of cancer cell behavior. Here, we will present some examples of how galectin-3 coordinates cellular responses in two critical hallmarks of cancer, tumor cell motility (see Galectin-3 Promotes Tumor Migration) and angiogenesis (see Galectin-3 in Tumor Vasculature).

Recently, Shetty and colleagues observed that galectin-3 promotes cell migration through its association with annexin A2 (AnxA2) on the plasma membrane of HER-2 negative breast cancer cells. This effect was abrogated by tunicamycin, an inhibitor of N-linked glycosylation, and chickpea lectin, a plant lectin highly specific for *N*-acetyl-d-galactosamine of AnxA2, showing that this migratory effect is dependent on galectin-3 CRD domain (115). To gain some insight regarding galectin-3 extracellular function on cell migration, Gao et al. (98) showed that exogenous galectin-3 promoted HeLa cell migration through the activation of ERK 1/2 *via* a calcium-sensitive and PKC-dependent pathway, which was not abrogated by endogenous galectin-3 knockdown. Moreover, the authors observed that this phenomenon was dependent on both CRD and N-terminal domain of galectin-3. In this context, a specific peptide targeting galectin-3 was able to inhibit prostate cancer cell migration, raising the possibility that galectin-3 inhibitors might avoid tumor spread (116).

Regarding galectin-3 migratory effect, it has been reported that galectin-3 silencing leads to a decrease in cell migration and invasion, through the regulation of different intracellular molecules. In osteosarcoma, this phenomenon was accompanied by a reduction in β-catenin expression and activation of important mediators of migration and invasion, such as FAK, Src, and Lyn (117). In human tongue cancer cell lines as well as pancreatic cell lines, the reduction of migration and invasion leads to a decrease in β-catenin, phospho-Akt, GSK-3β, and some MMP levels, indicating that galectin-3-mediated migration and invasion involves β-catenin degradation initiated by Akt phosphorylation in this model (118). In sarcoma cells, a similar trend was observed, as sarcoma cells devoid of galectin-3 were rendered more migratory when exposed to extracellular galectin-3, in a process dependent on the phosphorylation of AKT (100). Other study also indicated the correlation between urokinase-type plasminogen activator receptor (uPAR) levels and galectin-3-decreased migration in hepatocellular carcinoma cells (119). Likewise, Wu et al. (97) showed that overexpression of galectin-3 in colon cancer cells induced cell migration, which was correlated with lung colonization in a mouse model.

Again, in a metastatic murine melanoma cell line, B16F10, knocking down of galectin-3 reduced cell migration and invasion as well as MMP-1 levels (120). *In vivo*, low levels of galectin-3 in B16F10 cells caused a reduction in number of lung metastatic nodules. The authors investigated the molecular mechanism behind this biological effect and found that galectin-3 interacts with the transcriptional factor AP-1, promoting its binding to MMP-1 promoter driving the transcription of this metalloproteinase. In addition, it was also demonstrated that reduced levels of galectin-3 inhibited the binding of both transcription factors c-jun and fra-1 to the promoter sites of MMP-1. The interaction of intracellular galectin-3 and AP-1 was also described a year earlier in gastric cancer (121). In this study, the authors showed that the complex formed by galectin-3 and AP-1 binds to PAR-1 promoter driving its transcription, which was essential for galectin-3-mediated cell migration and invasion. Moreover, the role of MMP-1 in this phenomenon was pointed out, since its silencing caused a decrease in galectin-3-mediated cell migration.

Galectin-3 also regulates tumor cell migration through other mechanisms (Figure 1B). Exogenous galectin-3, through its ability to form lattices on the cell surface, restored FAK stabilization and cell motility in galectin-3 knockdown cancer cells (122). Lattice formation depends on the density of the glycosylated galectin-3 ligands found on the membrane and on the state of multimerization of galectin-3. Lattices form spatial plasma membrane subdomains, and together with lipid rafts and caveolin-dependent domains, are crucial for the spatial organization of signaling molecules. For example, galectin-3 and phosphorylated caveolin-1 act synergistically to promote EGF-induced RhoA activation and Mgat 5^+/+^ cell migration (123). The intracellular phosphorylated form of caveolin-1 activates focal adhesion kinase (84). Boscher and Nabi (123) observed that EGF promoted MDA-MB-231 breast cancer cell migration in a phosphocaveolin-1 and galectin-3-dependent manner, enabling the metastatic spread of these cells.

Additionally, it was demonstrated that the synergistic action of galectin-3 and caveolin-1 induced focal adhesion turnover and migration of differentiated thyroid cancer cells (124). Galectin-3 also promotes the raft-dependent endocytosis of integrins and plays a role in cell–matrix interaction (123). In this context, a novel function of galectin-3 has been reported related to the regulation of cell surface receptors by constitutive endocytosis. Specifically, Lakshminarayan and colleagues showed that both glycosphingolipids and extracellular galectin-3 mediate clathrin-independent carriers (CLICs)-dependent endocytosis. This work also reported that galectin-3 is critical for CD44 and β1-integrin endocytosis (125). In an intracellular context, Liu and collaborators reported that the absence of galectin-3 impairs keratinocyte migration, and this phenomenon was associated with the function of galectin-3 in the control of intracellular trafficking and cell surface expression of EGFR after EGF stimulation (126). Recently, another study demonstrated that galectin-3 knockdown potentiates the response to gefitinib (EGFR-tyrosine kinase inhibitor) treatment in esophageal squamous cancer cells, a type of cancer that overexpresses EGFR. The absence of galectin-3 impairs the EGFR endocytosis, which enhanced the antitumor effect in gefitinib-insensitive cells like cell viability, cell cycle, and invasion (127). Otherwise, Mazurek and collaborators observed that the silencing of galectin-3 restores tumor necrosis factor-related apoptosis-inducing ligand (TRAIL) sensitivity and promotes TRAIL-mediated endocytosis of TRAIL/death receptors. Thus, galectin-3 impairs TRAIL trafficking by anchoring them in cell membrane surface and blocks the execution of the apoptosis signal (128). Therefore, through regulation of endocytosis and intracellular trafficking of glycoproteins like integrins, galectin-3 assumes a novel role in the control of tumor cell adhesion and migration.

## Galectin-3 in Tumor Vasculature

The successful growth of a tumor mass – either a primary tumor or its metastasis – requires the establishment of an adequate blood supply, frequently, but not exclusively, achieved by the formation of new blood vessels. From premalignant to malignant lesions, the transition between an avascular to a vascular phase is required. Such transition is dictated by the balance of pro- and antiangiogenic signals. Therefore, the angiogenic switch activation requires the secretion of proangiogenic factors produced by both tumor and stromal cells within the tumor microenvironment (129).

Angiogenesis is characterized by a series of endothelial cell responses to this angiogenic balance, which include ECM degradation and budding, proliferation, migration, and tube formation of endothelial cells (130). Major signaling pathways orchestrating this process include VEGFR and fibroblastic growth factor receptor (FGFR) signaling pathways and Notch-dependent pathways (131–133). As mentioned earlier, extracellular galectin-3 is described to act at membrane proteins clusters through lattice formation (33, 134), promoting homotypic and heterotypic interactions and regulating substratum adhesion and receptor dynamics at the plasma membrane (135, 136).

Through its ability to form supramolecular structures through multimerization and through binding to glycoproteins that display multiple binding sites, galectin-3 serves as a scaffold protein that organizes signaling platforms on the cell surface of endothelial cells. In this sense, galectin-3 acts a cofactor responsible for modulating the angiogenic process, through amplification of the signal triggered by its partners in the context of the lattice formed on the cell surface. Galectin-3 binds directly on human umbilical vein endothelial cell (HUVEC) surface, amplifying the chemotactic response of these cells, besides the induction of capillary tube formation *in vitro* and angiogenesis *in vivo* (137). Galectin-3 is also required for the stabilization of epithelial–endothelial interaction networks, an essential process during angiogenesis (138). *In vivo*, endothelial cells present within tumors are enriched in galectin-3 ligands (13). Indeed, optimal vascularization of tumors was observed in model systems, where both tumor cells and stromal cells express galectin-3 (139). Markowska and colleagues demonstrated that galectin-3 promotes angiogenesis by interacting *via* CRD with complex *N*-glycans on αvβ3 integrin and through the activation of FAK-mediated signaling pathways that influence both VEGF and bFGF angiogenic activity. The same group also reported that galectin-3 contributes to the plasma membrane retention of VEGFR2, leading to increased angiogenic response to VEGF-A (42) (Figure 1C). A similar effect was observed when galectin-3 was used in combination with galectin-1 and both galectins retained VEGFR1 and VEGFR2 on the plasma membrane, enhancing endothelial cell growth and tube formation (140).

It is still a matter of debate to what extent the ability of galectin-3 to interfere with angiogenesis depends on its posttranslational processing. It is already known that galectin-3 collagen-like N-terminal domain is susceptible to extracellular cleavage *in vitro* and *in vivo* by MMP-2 and MMP-9 (60, 141). Cleaved galectin-3 displays ~20-fold higher affinity for endothelial cells (138), incrementing chemotaxis, invasion, and homotypic adhesion resulting in increased angiogenesis (142), as compared with the intact form of the lectin. The N-terminal domain is necessary for optimal oligomerization of galectin-3 (33, 143); however, it is conceivable that the collagen-like fraction retained after cleavage by MMPs is still sufficient for some degree of galectin-3 oligomerization.

Besides its direct role in endothelial cells, galectin-3 also influences migration of monocytes/macrophages (144) and their activation (145). Tumor-associated macrophages, which express galectin-3, have been shown to act in tumor angiogenesis and vessel maturation in a density- and phenotype-dependent manner (146, 147). Experiments performed by our group showed increased levels of active transforming growth factor β1 (TGFβ1) homodimer in galectin-3-expressing tumors (139). TGFβ1 is reported to induce chemotaxis and VEGF release by human monocytes/macrophages (148, 149). Bone marrow-derived macrophages (BMDMs) from galectin-3 KO mice showed a reduced basal secretion of VEGF when compared with BMDMs from WT mice. Upon TGFβ1 stimulation, WT-BMDMs secreted higher amounts of VEGF, as compared with KO-BMDMs (139). Galectin-3 targets not only macrophages but also platelets (Figure 1C), which release VEGF upon activation through a PKC-dependent pathway (150).

In addition to angiogenesis, galectin-3 has also been shown to participate in other process required to constitute the tumor vasculature, a process named vasculogenic mimicry. This process is characterized by the functional plasticity of aggressive tumor cells that behave as an endothelial cell in tumor vessels (151, 152). It has been proposed that galectin-3 expression in melanoma cells is necessary for their ability to form tube-like structures on collagen type I matrix. Gene expression signatures of galectin-3 deficient cells, as compared to their galectin-3-expressing parental cells, indicated co-expression of galectin-3 with a variety of endothelial cell markers that are involved in tumor angiogenesis, endothelial cell differentiation, and therefore in the process of vasculogenic mimicry (153). Understanding the mechanisms underlying this phenomenon will be necessary for future interventions.

## Concluding Remarks

Galectin-3 expression is dysregulated in different cancers. Its expression varies within different areas of tumors. For example, stressing conditions, such as hypoxia and nutrient deprivation, induce galectin-3 expression in breast cancer and glioblastoma. Under these conditions, accumulation of galectin-3 in the cytoplasm favors cell survival. Although the mechanistic details of this antiapoptotic activity of galectin-3 are not completely understood, there is accumulating evidence that galectin-3 tunes the different families of MAPKs. An interesting paradigm for galectin-3 function is the formation of nanoclusters with signaling molecules, such as K-RAS. In this context, galectin-3 serves as a scaffold protein, favoring the spatial organization of a signal transducer partitioned into the plasma membrane, where the interaction with other proteins is required for the efficient transduction of cellular signals. Secretion of galectin-3, either active through exosomes or other forms of protein release from the cytoplasm or passive through cell lysis or increased permeability of a dying cell, allows for novel functions of galectin-3, dependent on the extracellular ligand (growth factor receptors, integrins, and cadherins, among other glycoproteins). On the cell surface, the interaction of galectin-3 with glycoproteins illustrates the formation lectin lattices, another example of its scaffold function in the organization of supramolecular structures that interfere with receptor-triggered signal transduction. Extracellular galectin-3 is involved with cell migration (tumor cell, endothelial cell, and leukocyte migration) and the formation of vessels. At this level, secreted galectin-3 plays a homeostatic role in stressed microenvironments, as it induces formation of vessels that support normalization of both oxygen and nutrient delivery to tissues. In tumors, such homeostatic role would support tumor progression (Figure 1), and therefore it is a natural target for intervention. Galectin-3 is also an important modifier of immune cell function, which will constitute now another layer of complexity in its roles in cancer progression, which will be dealt with in other reviews in this series.

## Author Contributions

AC, LA, SB, and RC contributed in the idealization of the topic, critical reading of the references, discussion, and writing.

## Conflict of Interest Statement

The authors declare that the research was conducted in the absence of any commercial or financial relationships that could be construed as a potential conflict of interest.
